# The latest developments in the area of therapeutic delivery excluding some diseases, such as COVID-19 and the big three (HIV/AID, malaria and tuberculosis)

**DOI:** 10.4155/tde-2021-0066

**Published:** 2021-10-21

**Authors:** Catarina Pinto Reis

**Affiliations:** ^1^iMED, ULisboa, Research Institute for Medicines, Faculty of Pharmacy, Universidade de Lisboa, Av. Prof. Gama Pinto, 1649-003 Lisboa, Portugal; ^2^IBEB, Biophysics & Biomedical Engineering, Faculty of Sciences, Universidade de Lisboa, Campo Grande, 1749-016, Lisboa, Portugal

**Keywords:** cancer, cardiovascular diseases, flavonoids, gut microbioma, new approvals, nutrition and health, orphan medicines, patents, Pompe disease, rare diseases, research and development, severe atopic dermatitis, severe plaque psoriasis, systemic lupus erythematosus, von Hippel-Lindau disease, Waldenström's macroglobulinemia, Wilson disease

## Abstract

It is the beginning of a new decade, where COVID-19 and the ‘big three’ (HIV/AID, malaria and tuberculosis) have raised public awareness of the type of challenges researchers face every day. Beyond these diseases, there are also still many more for which scientists are working to develop new therapies and their impact in healthcare is enormous too. This industry update covers the period 1–31 August 2021, and some examples of research and approvals for many other diseases excluding COVID-19 and the ‘big three’ are presented. There is a progressive trend of approvals of novel drug candidates and the proposal of new indications for the existing ones. Some patents related to rare diseases were also published during this month. Information and analyses were sourced from scientific literature, regulatory and patent agencies, websites and press releases of the companies (and not based upon personal opinion). The main reason of leaving COVID-19 research outside the scope of this update is mainly due to the rapid growth and change in this field; some preliminary results require further research and scientists must be aware of the final impact that this research could have on public opinion. Moreover, as a result of disruptions to health care in the face of COVID-19, several research groups simply stopped their research in other diseases and, for that reason, it is imperative to sum up some important advances in other critical diseases and health areas.

## Research & development

One important research area is Nutrition; the linking of the two fields of nutrition and health has been increasingly recognized as relevant during this pandemic scenario [1]. According to some previous data, it has been reported that the double burdens of malnutrition (undernutrition and obesity) seem to be significant risk factors for illness, which consequently leads to new challenges in finding effective and safe therapies related diseases.

## Gut bacteria & flavonoid-rich foods are linked to better health

On 23^rd^ of August, researchers found that gut bacteria and flavonoid-rich foods are linked in terms of blood pressure regulation [2]. In this study, researchers evaluated the association of consumption of flavonoid-rich foods with blood pressure and gut microbiome diversity. Gut microbiota is highly variable between individuals. Herein, the focus was on specific (not all) foods rich in flavonoids.

Flavonoids are natural compounds that can be found in vegetables, fruits and plant-based foods. Tea, chocolate and wine are some examples of important sources of flavonoids. Flavonoids are broken down by the gut microbiome.

A sample of 904 adults (age range between 25 and 82 years) was included in this study. Participants were evaluated at regular follow-up examinations in terms of food intake (calculated from a self-reported questionnaire), gut microbiome and blood pressure levels. Lifestyle factors, as well as family history related to cardiovascular diseases, were considered in this study. In terms of the methodologies used, analysis of flavonoid values was assigned following United States Department of Agriculture Recommendations, while gut microbiomes were assessed *via* fecal bacterial DNA extracted from stool samples. After an overnight fast, the blood pressure levels of the participants were measured in triplicate using 3-min intervals.

This study reported that a high intake of flavonoid-rich foods was linked to lower systolic blood pressure levels. The same relationship between the intake of flavonoid-rich foods and diversity in gut microbiome was observed; up to 15.2% of this association could be explained by considering the diversity found in gut microbiome. As a cited example and according to the authors, eating 1.6 servings of berries per day was associated with an average reduction in systolic blood pressure levels of 4.1 mm Hg. Finally, about 12% of the association was explained by gut microbiome factors.

According the authors, future trials should clarify the metabolic profile. It is important to address more accurately each role in regulating the consequences of flavonoid intake on blood pressure. These findings can be considered as very promising ones. Thus, this effect should be tested in a very near future, probably by using it in association with commonly used hypertension drugs, by using new drug candidates or even by combining it with encapsulated existent or new drug in delivery systems in order to optimize this hypotensor effect. In any case, although these associations are still unknown, they are suggestive of potential approaches to generating very inspiring results.

## Advances in cancer research

Cancer is a leading cause of death worldwide, and it accounted for nearly 10 million deaths in 2020 [3]. During the COVID-19 pandemic, millions of people opted to skip their screening appointments, mostly of them delaying a proper diagnosis. According to the American Cancer Society and National Comprehensive Cancer Network, an estimated 22 million cancer screenings were lost or canceled between March and June of last year, resulting in decreases in screening rates (94% overall, according to Epic Health Research Network) for breast, colon and cervical cancers [4].

About new advances in Cancer Research, the University of British Columbia's and BC Cancer Research Institute have discovered a weakness in a key enzyme that solid cancer cells rely on to adapt and survive in hypoxia [5].

It is known that the microenvironment of solid tumors is heterogeneous in terms of oxygen availability. Most solid tumors contain regions of hypoxia, and this low-oxygen environment leads to a shift in carbon usage, increasing dependence on glycolysis for energy production. It also results in the accumulation of acidic by-products and a reliance upon pH regulatory enzymes and transporters to maintain an alkaline intracellular pH.

To overcome this fact, cells generally tend to adapt by releasing enzymes that neutralize the acidic conditions. With this, cells not only survive, but ultimately become more aggressive, easily spreading to other organs. One of the enzymes involved in this mechanism is called Carbonic Anhydrase IX.

In this study, authors identified a redox homeostasis network containing the iron-sulfur cluster enzyme, NFS1, that can be crucial for the therapy of solid tumors. Depletion of NFS1 or blocking cyst(e)ine availability while targeting Carbonic Anhydrase IX enhanced ferroptosis and significantly inhibited tumor growth. Thus, authors found that an alkaline intracellular pH seems to play a critical role in suppressing ferroptosis.

This last finding may help the development of innovative therapeutic strategies for solid tumors to overcome hypoxia- and acidosis-mediated tumor progression, as well as the development of innovative strategies against drug resistance commonly observed against numerous antitumoral drugs.

## Risk factors of numerous cancers

The World Cancer Research Fund Third Expert Report concluded that diet and nutrition, including obesity and low physical activity, are risk factors for several cancers. One recent study based on 860 reviews (meta-analyses) investigated the link between food and nutrient intake and the potential risk of either developing or dying from 11 types of cancers [6]. Authors found that coffee consumption was associated with a lower risk of developing liver cancer and basal cell carcinoma of the skin. The precise mechanism is still under discussion, but it seems that this effect can be connected to its antioxidant and anti-inflammatory properties. Both properties might protect against several diseases triggered by inflammation, like cancer. In contrast, a completely different observation was seen with alcohol intake. Mechanistically, when alcohol is metabolized, the by-products can bind to DNA, resulting in mutations that could become cancerous. Besides this effect, alcohol can also increase the levels of the hormones linked to the development of some types of cancer (e.g., breast cancer).

Further research must be done in order to better understand the exact mechanisms. Nevertheless, this finding can be potentially helpful when considered in association with a specific drug, and in treating several diseases such as cancer, inflammation or other.

## New approvals

Bristol Myers Squibb has announced that the European Commission (EC) has approved Opdivo (nivolumab) for the adjuvant treatment of adult patients with esophageal or gastroesophageal junction cancer who have residual pathologic disease following prior neoadjuvant chemoradiotherapy [7]. Opdivo also received approval from the US FDA in May 2021. Gastroesophageal junction adenocarcinoma is a type of cancer of the esophagus. The exact definition has been an area of controversy [8]. Gastroesophageal junction tumors includes distal esophageal cancers, proximal gastric cancers and cancers of cardia. This heterogeneity results in divergences regarding their categorization, pathophysiology and therapeutic approaches. Opdivo's approval was based on results of the Phase III CheckMate-577 trial. This study demonstrated that treatment with the drug following neoadjuvant chemoradiotherapy and complete surgical resection doubled the primary end point of disease-free survival when compared with placebo. The safety of nivolumab was coherent with earlier studies.

Another successful example is related to anifrolumab. US FDA has approved AstraZeneca's Saphnelo (anifrolumab) for the treatment of moderate to severe systemic lupus erythematosus [9]. By the classic definition, systemic lupus erythematosus is an autoimmune disorder characterized by antibodies to nuclear and cytoplasmic antigens, multisystem inflammation, protean clinical manifestations, a relapsing/remitting course. These patients often have a long-term organ damage as well as poor health-related quality of life. With this approval, Saphnelo becomes the first type I interferon (type I IFN) receptor agonist after AZ's Saphnelo clinical development programme (TULIP Phase III and MUSE Phase II trials). According to these two studies, more patients receiving Saphnelo treatment had a reduction in overall disease activity in their skin and joints, and also achieved a reduction in oral corticosteroid use when compared with a placebo.

The Scottish Medicines Consortium, for use via NHS Scotland, approved Bavencio (avelumab) as a first-line maintenance treatment for adult patients with locally advanced or metastatic urothelial carcinoma (bladder cancer) that has not progressed following platinum-based chemotherapy [10]. Bladder cancer is the tenth most common cancer worldwide [11]. Platinum-based chemotherapy is currently one of the first-line treatment for patients with advanced disease. However, most patients have a disease progression within 9 months of initiation of treatment. Prognostic of patients with metastatic disease is very poor; only 5% of them will live longer than 5 years. Herein, avelumab is the first immunotherapy to be approved as a maintenance treatment for eligible bladder cancer patients by Scottish Medicines Consortium. The JAVELIN Bladder 100 study (Phase III) demonstrated a 7.1-month improvement in median overall survival with avelumab as first-line maintenance *plus* best supportive care compared with best supportive care alone. It also showed a 31% of reduction in the risk of death.

For Pompe's disease, a rare disease with around 1 case in 40,000 births, there are also good news, in that the FDA has approved Nexviazyme (avalglucosidase alfa-ngpt) [12]. Pompe disease is caused by mutations (up to 300 different mutations) in the *GAA* gene. *GAA* is a gene responsible for an enzyme called acid alpha-glucosidase (GAA). This enzyme is involved in the breakdown of glycogen in lysosomes. Here, glycogen is converted by this enzyme into glucose. In Pompe's disease, heart and skeletal muscles cells are the most particularly affected. The age of onset and severity of Pompe disease are generally correlated to the degree of enzyme deficiency. Nexviazyme is an enzyme replacement therapy which it is involved in reduction of glycogen. The effectiveness of Nexviazyme was demonstrated in 100 patients. The FDA granted this application with Fast Track, Priority Review and Breakthrough Therapy designations. In regulatory area, Nexviazyme also received an Orphan Drug Designation.

On August 13, FDA approved Welireg (belzutifan). This drug is a hypoxia-inducible factor inhibitor for patients with von Hippel-Lindau disease who require therapy for associated renal cell carcinoma, CNS hemangioblastomas or pancreatic neuroendocrine tumors, not requiring immediate surgery [13]. von Hippel-Lindau disease is a rare genetic disease which puts patients at risk of developing benign blood vessel tumors as well as several cancers such as renal cell carcinoma. Up to 70% of people with this rare disease develop the latter type of cancer. Belzutifan was investigated in an open-label clinical trial in 61 patients. Patients had von Hippel-Lindau-associated renal cell carcinoma diagnosed and at least one measurable solid tumor localized to the kidney. In this group, an overall response rate of 49% was reported. All patients were followed for a minimum of 18 months. The median duration of response was not reached; 56% of responders had duration of response of more than 12 months and a median time to response of 8 months. The other enrolled group of patients had von Hippel-Lindau-associated tumors with central nervous system hemangioblastomas or pancreatic neuroendocrine tumors. In this first sub-group of patients (n = 24), in other words, with central nervous system hemangioblastomas, patients showed an overall response rate of 63%. The median duration of response was not reached with 73% of patients having response duration for more than 12 months. On the other second sub-group (n = 12), in other words, with pancreatic neuroendocrine tumors, patients had an overall response rate around 83%. The median duration of response was also not reached, with 50% of patients having response duration for more than 12 months.

Another approval was recently announced by Vivet Therapeutics and Pfizer Inc. (NYSE: PFE) [14]. The FDA has granted Fast Track designation to VTX-801: gene therapy for the treatment of Wilson's Disease. The safety, tolerability and pharmacological activity of this novel gene therapy will be evaluated in a Phase I/II clinical trial. Wilson disease is an inherited autosomal recessive disorder of copper balance, leading to hepatic damage and neurological disturbances [15]. The worldwide prevalence is estimated to be 1 in 30,000 or even higher in certain populations with consanguinity. It is caused by mutations in the gene encoding the ATP7B protein. This mutation reduces the ability of the liver to control copper levels. This observation can also occur in other tissues. These levels of copper will cause severe hepatic damage, neurological symptoms and, potentially, death. VTX-801 has been granted Orphan Drug Designation by FDA and by EC but also as Fast Track designation by the FDA. This medicine aims to restore copper homeostasis. Generically, it is based on a replication-deficient recombinant adeno-associated viral vector (rAAV) consisting of an AAV liver tropic capsid containing a single-stranded DNA genome carrying a shortened version of the *ATP7B* gene (*ATP7B-minigene*).

According to the recent press release of Boehringer Ingelheim [16], Jardiance (empagliflozin) has been approved by the FDA. This medicine aims to reduce the risk of cardiovascular death and hospitalization for adults with heart failure with reduced ejection fraction. The EMPEROR-Reduced Phase III trial evaluated the effect of adding Jardiance 10 mg *versus* placebo to standard of care in 3730 adults with and without Type 2 diabetes who had heart failure (functional class II, III or IV) and a left ventricular ejection fraction of 40% or less. In this study, Jardiance reduced the relative risk of the primary composite end-point of time to cardiovascular death or hospitalization for heart failure by 25% (5.3% absolute risk reduction) *versus* placebo.

Another example of a recent approval is a drug for severe plaque psoriasis. EC has approved UCB's Bimzelx for the treatment of moderate to severe plaque psoriasis in adults [17]. Bimekizumab is a monoclonal antibody designed to attach to interleukins (IL-17A, IL-17F and IL-17AF). High levels of these interleukins have been related to inflammatory diseases. Thus, Bimekizumab prevents interleukins from interacting with their receptors on the surface of the epidermis, which reduces inflammation. The approval was supported by three Phase III trials where Bimzelx demonstrated higher levels of skin clearance compared with placebo, ustekinumab and adalimumab. About 60% of bimekizumab-treated patients (test group) achieved complete skin clearance at week 16. In addition, this effect was maintained for up to a year. Bimzelx is currently under FDA review, and regulatory reviews are also underway in Great Britain, Australia, Canada and Japan.

On 25 August 2021 and according the press release of Abbvie's [18], EC has approved JAK inhibitor Rinvoq (upadacitinib). This medicine is indicated for the treatment of moderate-to-severe atopic dermatitis in adults and adolescents 12 years and older who are candidates for systemic therapy. This approval was supported by data from a large Phase III trial investigating Rinvoq monotherapy or with topical corticosteroids in 2500 adults and adolescents. Rinvoq met primary and secondary end-points, demonstrating an improvement in skin clearance and itch reduction compared with placebo.

On 31 August 2021, the FDA approved zanubrutinib (Brukinsa, BeiGene) for adult patients with Waldenström's macroglobulinemia [19]. Waldenström macroglobulinemia is a lymphoproliferative disease of B lymphocytes. This disease is characterized by a lymphoplasmocytic lymphoma in the bone marrow and by IgM monoclonal hypergammaglobulinemia [20]. It was first described in 1944 by Jan Gösta Waldenström. This approval was based on a non-comparative assessment of response but also duration of response from the zanubrutinib arms.

## New R&D collaborations & some market changes

A multi-year research collaboration related to discovery, development of several modalities (antibodies, small molecules, etc.) and commercialization was recently announced by Eli Lilly and Company (NYSE: LLY) and Lycia Therapeutics, Inc. The platform that will be used in this research is LYTAC, and concerns the development of new therapies for cancer and autoimmune diseases [21].

Also in this month, in terms of acquisitions, Bayer AG acquired Vividion Therapeutics, Inc. (Vividion) [22]. Vividion Therapeutics has been working in multiple precision oncology and precision immunology targets. One of these promising examples is the transcription factor NRF2 antagonist for the potential treatment of NRF2 mutant cancers. Another example of their research area is connected to NRF2 activators for various inflammatory diseases - among other preclinical programs.

## Examples of new patents

On 3^rd^ of August, Adamas Pharma, LLC (CA, USA) was granted with US Patent about amantadine compositions [23]. This drug is generally indicated for the treatment of idiopathic Parkinson's disease, post-encephalitic Parkinsonism and Symptomatic Parkinsonism followed by carbon monoxide intoxication, among other applications. The effectiveness measures for once nightly administration of the proposed amantadine oral compositions were higher than the current available formulations of amantadine (immediate release forms) which are administered in different doses. Additionally, these new amantadine formulations provide improved relative tolerability, particularly when administered once daily and especially when once daily administered.

Eisai R&D Management Co., Ltd. (Tokyo, JP) was granted with a US Patent on 10^th^ of August. This patent describes a new liposomal formulation with eribulin and a PD-1 antagonist [24]. This patent only described preclinical studies but the further research involving nanotechnologies is very optimistic, like many other studies in several fields of research [25,26]. The target tumor was selected from several types of cancers. They focused their results on breast cancer, colorectal cancer and kidney cancer.

On 17 August 2021, Genzyme Corporation (MA, USA) was granted with a US Patent no. 11,091,759 [27]. This patent described new method using iRNA compositions for treating some disorders (e.g. bleeding or hemophilia) that would take advantage from blocking or decreasing the expression of a *Serpinc1* gene. Specifically, *Serpinc1* inhibits thrombin as well as coagulation factors (X, IX, XI, XII and VII). Consequently, it regulates the blood coagulation cascade. It is known that the activity of *Serpinc1* is improved by the existence of heparin and other related glycosaminoglycans. Concerning hemophilia, there is no cure for this disease. It has been reported that it can be regulated with the deficient clotting factors; unfortunately, it is observed that certain patients develop antibodies and thus they become refractory to that treatment. Consequently, the development of high-titer inhibitors to factor VIII and other coagulation factors is still a great challenge. Currently, the common therapeutic strategies are based on the use of factor eight inhibitor bypass activity and activated recombinant factor VII, continuous factor replacement, plasmapheresis and, ultimately, immune tolerance therapy. Accordingly, none of the previous strategies are entirely effective. The present invention is based on the application of very low doses (e.g., doses at least about 30 times lower than common doses) of a GalNAc linked double stranded RNAi agent which inhibits the expression of *Serpinc1* in terms of potency and duration.

On same day, The Trustees of the University of Pennsylvania (PA, USA) was granted with a US Patent no. 11,090,392 for a new therapy for choroideremia [28]. This disease is an X-linked retinal degeneration and it progresses through mid-life. This disease is a positive target for gene therapy due to loss of function of a protein necessary for retinal cell health: Rab Escort Protein 1. This protein is encoded by the choroideremia gene. The Trustees of the University of Pennsylvania propose a novel approach where the *choroideremia cDNA* is packaged in recombinant adeno-associated virus. It is believed that this therapy can increase the efficacy and safety, since a lower dose is used. It is also expected that this transgene cassette could theoretically maximize the level of production of the experimental protein in comparison to the levels produced by using the endogenous sequence.

NOVARTIS AG (Basel, Switzerland) was granted with US patent on 24^th^ of August. This patent comprises a method for reducing the treatment burden for patients who have an intraocular neovascular disorder. It comprises the administration of a VEGF antagonist on a specific dosing schedule (treatment intervals of 8 and/or 12 weeks) [29]. An example of a disease that can be treated using this technology is the age-related macular degeneration. This disease is the leading cause of severe vision loss, affecting 10–13% of individuals over the age of 65 years. The etiology is complex. It seems that VEGF is elevated in this ocular degeneration, playing a key role in the neovascularization process.

Another patent, US Patent n.° 11,103,495, which the assignee is TRIS PHARMA, INC. (NJ, USA) was published on 31^st^ August and provides an oral methylphenidate extended release tablets with a 12-h extended release profile [30]. These novel tablets contain an uncoated and a coated methylphenidate-ion exchange resin complex and an uncomplexed methylphenidate as a drug.

As a summary, several examples of success were described in this update. It is widely accepted there is still a need for further research to fully understand specific diseases, provide insight into their pathogenesis, or even reveal novel drug targets or repurpose existing drugs for different applications. It is clear that only with collective efforts, investment and sharing of scientific knowledge is it possible to provide the opportunity to more effectively treat both common and rare diseases, benefitting all players: researchers from academia or industry, physicians, pharmacists and other health professionals, but the most important, the patients.
